# Genetic Distinctiveness of the Herdwick Sheep Breed and Two Other Locally Adapted Hill Breeds of the UK

**DOI:** 10.1371/journal.pone.0087823

**Published:** 2014-01-29

**Authors:** Dianna Bowles, Amanda Carson, Peter Isaac

**Affiliations:** 1 Department of Biology, University of York, York, United Kingdom; 2 The Sheep Trust, registered charity 1094514, University of York, York, United Kingdom; 3 IDna Genetics Ltd, Norwich Research Park, Norwich, United Kingdom; CSIRO, Australia

## Abstract

There is considerable interest in locally adapted breeds of livestock as reservoirs of genetic diversity that may provide important fitness traits for future use in agriculture. In marginal areas, these animals contribute to food security and extract value from land unsuitable for other systems of farming. In England, close to 50% of the national sheep flock is farmed on grassland designated as disadvantaged areas for agricultural production. Many of these areas are in the uplands, where some native breeds of sheep continue to be commercially farmed only in highly localised geographical regions to which they are adapted. This study focuses on three of these breeds, selected for their adaptation to near identical environments and their geographical concentration in regions close to one another. Our objective has been to use retrotyping, microsatellites and single nucleotide polymorphisms to explore the origins of the breeds and whether, despite their similar adaptations and proximity, they are genetically distinctive. We find the three breeds each have a surprisingly different pattern of retrovirus insertions into their genomes compared with one another and with other UK breeds. Uniquely, there is a high incidence of the R0 retrotype in the Herdwick population, characteristic of a primitive genome found previously in very few breeds worldwide and none in the UK mainland. The Herdwick and Rough Fells carry two rare retroviral insertion events, common only in Texels, suggesting sheep populations in the northern uplands have a historical association with the original pin-tail sheep of Texel Island. Microsatellite data and analyses of SNPs associated with *RXFP2* (horn traits) and *PRLR* (reproductive performance traits) also distinguished the three breeds. Significantly, an SNP linked to *TMEM154*, a locus controlling susceptibility to infection by Maedi-Visna, indicated that all three native hill breeds have a lower than average risk of infection to the lentivirus.

## Introduction

The genetics and conservation of locally adapted livestock breeds is attracting considerable interest currently [1]–[3]. This interest arises in part from the significant contribution of the breeds to food security in marginal land areas of the world unsuitable for other means of agricultural production. Also, the ability of the breeds to adapt and thrive under what are often harsh environmental conditions implies they may have adaptive fitness traits that could become increasingly useful to the future sustainability of mainstream agriculture.

Of importance to the global and national conservation strategies of these breeds is their genetic characterization, confirmation that they are genetically distinctive and an assessment of the biodiversity each breed may contribute to future trait selection [4]–[6].

In this context, the UK has a substantial number of native sheep breeds [7]. Many of these are now numerically scarce and largely maintained in small numbers by breeders committed to conservation of rare, endangered livestock. There is however another category of native sheep breeds that are not rare, are commercially farmed and continue to exist in tens of thousands [7]. These are also recognized to be at risk, but for the reason that they are highly concentrated in specific geographical regions to which they are adapted [8].

Amongst these locally adapted breeds are the hill breeds of the northern uplands. These typically are farmed on Disadvantaged or Severely Disadvantaged Areas for agricultural production [9] and are characterized by their ability to thrive in the harsh environments of mountains and moorlands, rearing lambs on low inputs of feed and management.

The endangerment of breeds such as these was first recognized in the UK during the Foot and Mouth Disease (FMD) epidemic of 2001 which started in the northwest of England and disproportionately affected livestock farmed in that region. Losses to the Herdwick sheep breed were of particular concern, which led to the setting up of the first national gene bank for sheep and a government commitment to survey the level of geographical concentration that existed amongst commercially farmed regional breeds. Working with Sheep Breeder Associations (SBAs) and more than 1000 breeders, individual flocks of 12 regional breeds were geo-referenced [8]. The data showed certain of these ‘Heritage’ breeds were indeed extremely concentrated with for example, up to 95% of the Herdwicks numbering some 47 000 animals, tightly clustered within 23 km of the breed's mean centre in the Lake District National Park.

**Figure 1:**
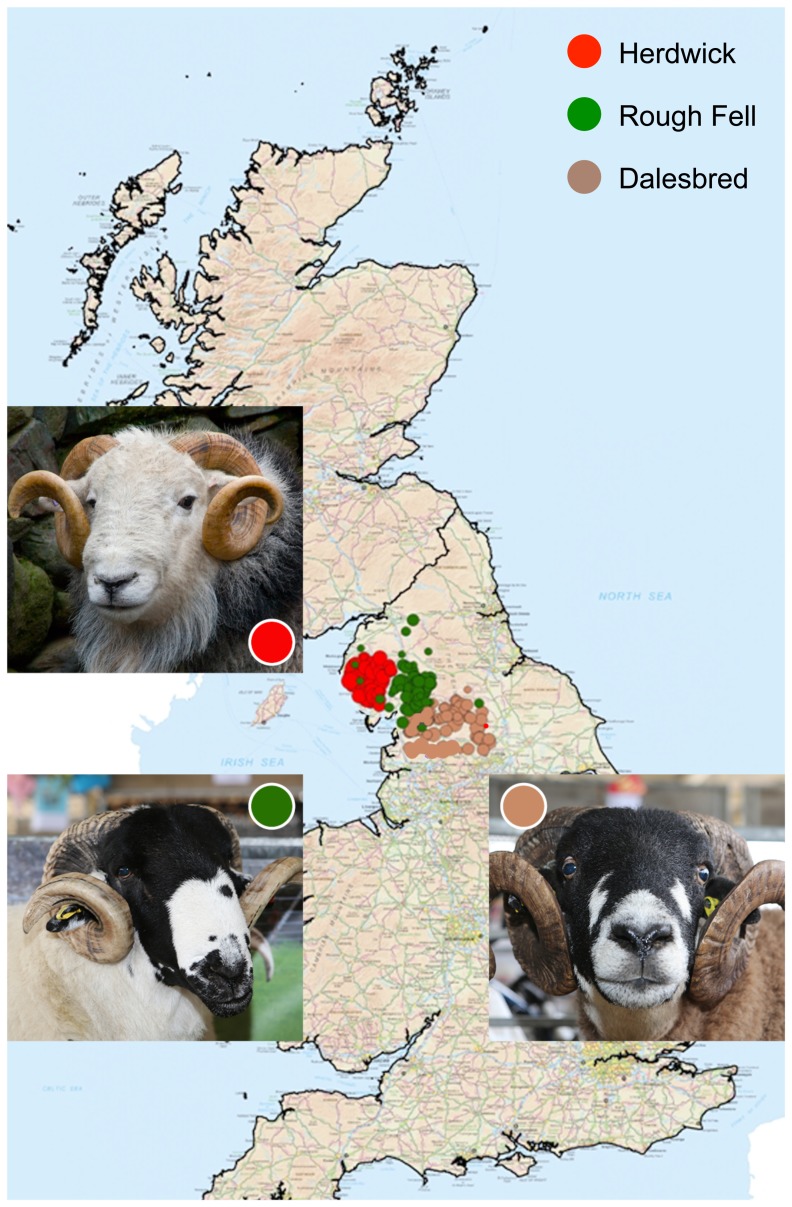
The distribution of three locally adapted sheep breeds of the UK uplands and their geographical concentration shown on a composite map using data described in [8].

A significant issue for prioritising conservation strategies is whether breeds concentrated in proximate geographical regions and locally adapted to very similar environmental conditions are genetically distinctive. We have selected three breeds from the northern uplands to explore this possibility. Their distribution is illustrated in Figure 1, together with an image of a typical ram from each breed. The Herdwick is an iconic image of the Lake District; Rough Fells are farmed in partially overlapping regions in Cumbria to the east of the Herdwicks and the distribution of the Dalesbred is to the south and further east into Lancashire and Yorkshire. In each region, the sheep breed continues to be valued for its cultural heritage and contribution to local economic sustainability [10]–[12].

There have been no previous molecular genetic analyses of these three breeds. Large scale European studies by the Econogene Consortium and others [13]–[16] involved some UK breeds, including the Exmoor Horn and Welsh Mountain shown subsequently to be geographically concentrated [8]. Isolated Scottish Island populations have also been studied in global surveys, including the numerically scarce breed of the Soay, and those in the Hebrides, Faeroe and Orkney [15], [17]–[19].

There have been studies specifically focused on geographical proximate breeds elsewhere in Europe, including such as those of Northern Spain [20], [21], Northern Europe, the Baltic and the Volga region of the Russian Federation [22]–[24].

We have used a combination of approaches to study the genetic distinctiveness of the Herdwick, Rough Fell and Dalesbred. Each approach has been used widely in previous studies of sheep breeds across the world, revealing new information about the history of sheep domestication, relatedness of populations and increasingly, the genes encoding interesting phenotypes and important production traits in livestock [16]–[18], [22], [25]–[29].

In total, our results show a considerable level of genetic distinctiveness amongst the three northern hill breeds. We provide evidence that the Herdwick has retained characteristics of a primitive genome and together with Rough Fell and Dalesbred can be distinguished from one another and other UK breeds studied previously. The findings emphasise the importance of conservation strategies to protect their genetic resources.

## Materials and Methods

### Materials, ethics statement and sampling

Our study has involved analyzing equal numbers of registered breeding males (rams) from each breed. Through collaboration with the respective Sheep Breeders Associations (SBAs) and individual breeders we were able to ensure samples were each obtained from different commercial flocks of known provenance and breeders who could confirm the familial origins and unrelatedness of the rams selected. Typically, a ram will sire up to 150 progeny of lambs each year over 6 to 10 years, compared to a ewe that will produce up to two offspring per year over a shorter breeding life. As a consequence, rams contribute a major genetic resource to the breed but represent a potential risk to diversity as they form only a numerically small fraction of the total population. Also, such as for the Herdwick breed, not all SBAs register females, so that animals conforming to breed standard phenotypes are only formally recorded if they are males, and sampling from a flock may lead to many related animals. None of the genetic markers we used were linked to sex chromosomes.

Blood spots were collected onto FTA cards from individual rams by their breeders using approved guidelines as part of a routine prion gene surveillance programme in collaboration with Innovis. Innovis provided the project with blood blots subsequent to their use for prion genotyping. DNA was extracted from blood blots of 19 rams per breed as described in [30], using duplicate 2 mm discs taken from the FTA cards. We have used three different approaches to explore the genetic resources of the breeds, including their retrotypes, single nucleotide polymorphisms (SNPs) known to be associated with phenotypes and a subset of microsatellites.

### Retrotype analysis

Retrotypes were analysed as described in [17], [31], and details of the procedures together with the raw data on each insertion of an endogenous Jaagsiekte sheep retrovirus (enJSRV) are given in File S1 and Table S1. As in [17], we scored an insertion event as dominant and therefore a positive score could either be homozygous or heterozygous for the enJSRV.

### SNP analysis

Three SNP loci were analysed, one linked to *TMEM*154 [25], [26] associated with reduced ovine Lentivirus susceptibility; a second linked to the gene encoding the Prolactin receptor protein (PRLR), associated with a variety of reproductive traits [27], [28], and a third linked to the gene encoding the Relaxin/insulin-like family peptide receptor 2 (RXFP2) associated with horn traits [19], [29]. The details of the SNP loci, primers, procedures and genotypes are given in File S1 and Table S2.

### Microsatellite genotyping

The details of the microsatellite markers selected from [16], [23], primers, procedures and raw data sets are provided in File S1 and Table S3. The molecular data from use of the microsatellites was analysed using Structure version 2.3.4 [32] for 1 to 6 populations, with a burn-in period of 1000000 iterations, and data collection period of 1000000 iterations. The most likely number of populations in the dataset, independent of breed affiliation was for K = 3 (−1525.1). F-statistics (*F*_ST_) were computed using FSTAT 2.932 [33], and population subdivision was examined using Weir and Cockerham's unbiased estimator of Wright's fixation index [34], [35]. Inbreeding coefficients (*F*_IS_) were calculated per marker per breed as shown in Table S4.

## Results and Discussion

The aim of the study has been to use a range of approaches to gain information on the potential common origins of the three breeds and whether there is evidence to suggest they are genetically distinctive.

### Analysis of retrotypes

Recent studies have shown that endogenous retroviruses, related to the Jaagsiekte sheep retrovirus (enJSRVs) are useful genetic markers to study the historical origins of domestic sheep breeds across the world [17]. Whilst most enJSRVs loci were found to be fixed in domestic sheep, others were insertionally polymorphic [31]. As Chessa *et al* [17] reasoned, since each endogenous retrovirus in the host genome arises from a single irreversible integration event, populations sharing the retrovirus DNA in the same genomic location must be related phylogenetically.

**Figure 2:**
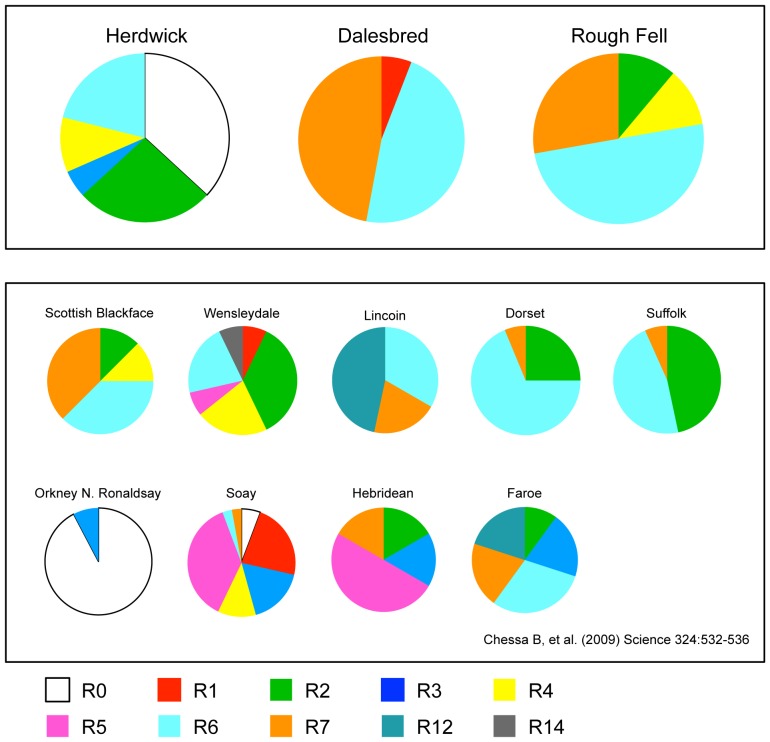
Analysis of retrotypes. The four most common retrovirus events (enJSRV-18, enJSRV-7, enJS5F16, enJSRV-8) were used by Chessa *et al* [17] to construct a classification scheme based on retrotypes. The pie charts show the relative proportion of retrotypes in each of the three hill breeds in this study. For ease of comparison, the colouring of each retrotype is the same as used in [17], and pie charts of example breeds from [17] are also illustrated in the Figure.

The presence or absence of the six enJSRVs used in [17] were assayed in genomic DNA of individuals from the three breeds. The data for the four common retrovirus insertions are shown in Figure 2, given as the retrotype pattern of each breed. For ease of comparison, the retrotype classification devised in [17] and based on the combinations of the four common enJSRVs in a population is shown in Table 1. The raw data, expressed as the number of positive samples for each of the six enJSRVs assayed per individual for each of the three breeds is provided in Table S1; this also provides the presence and distribution of the two rare insertion events of enJSRV-15 and enSRV-16 that due to their extreme rarity were not included by Chessa and coworkers in the retrotype classification scheme.

**Table 1:** The retrotype classification defined in [17] and based on the combination of the four common retroviral insertions in a population is shown in the Table, ‘present’ is highlighted in bold style font.

Retrotype	enJSRV-18	enJSRV-7	enJS5F16	enJSRV-8
R0	Absent	Absent	Absent	Absent
R1	Absent	**Present**	Absent	Absent
R2	**Present**	Absent	Absent	Absent
R3	Absent	Absent	**Present**	Absent
R4	**Present**	**Present**	Absent	Absent
R5	Absent	**Present**	**Present**	Absent
R6	**Present**	Absent	**Present**	Absent
R7	**Present**	**Present**	**Present**	Absent
R8	Absent	Absent	Absent	**Present**
R9	Absent	Absent	**Present**	**Present**
R10	Absent	**Present**	**Present**	**Present**
R11	**Present**	Absent	Absent	**Present**
R12	**Present**	Absent	**Present**	**Present**
R13	**Present**	**Present**	Absent	**Present**
R14	**Present**	**Present**	**Present**	**Present**

As shown in Figure 2, a unique feature of the Herdwick compared with the other two upland breeds in this study is the high proportion of the R0 retrotype in the rams sampled. This lack of enJSRVs was suggested to be a feature of primitive genomes, described in [17] as relics of first migrations originating from the earliest occurrence of domesticated sheep. The Herdwick population is likely to have remained relatively isolated, given that exposure to other breeds carrying enJSRVs would have led to an increased presence of retrovirus insertions.

In the global study of 133 breeds, the R0 retrotype was detected to any abundance in only 10 breeds world-wide [17]. No R0's were identified in the UK mainland breeds, although only one northern upland breed was analysed in that study, the Scottish Blackface. To provide a ready baseline for comparison, pie charts taken from [17] of five mainland breeds (one Upland, two Longwools and two Downs) are reproduced in Figure 2, together with those of four breeds from the Northern Islands of the UK. In the context of the R0 retrotype we found in the Herdwick population, it can be seen that only the North Ronaldsay of Orkney has that retrotype, but to an extreme proportion.

Other breeds exhibiting substantial levels of the R0 retrotype were located outside the UK, and in addition to Mouflon orientalis, were populations in Sweden (Rya, Gotland, Gute), Finland (Finnsheep, Kainuu Grey Sheep, Aland) and Iceland (Icelandic, Leader sheep) [17]. Our data suggest that the Herdwick may originate from a common ancestral founder flock to these other breeds.

Interestingly, the data analyses of Chessa *et al* revealed a primitive group of peripheral Northern European breeds, characterized by the absence of enJSR-18 and/or a high proportion of enJSRV-7, consisting of the Soay, Hebrides, Orkney, Icelandic and Nordic breeds [17]. Whilst the lack of insertionally polymorphic enJSRVs in the Herdwick population indicates a primitive feature, the breed however can be distinguished from the generic group, since >50% of the breed sample carried enJSRV-18 (within R2, R4, R6 as defined in Table 1) and very few carried enJSRV-7. Sample sizes in [17] for each of the populations discussed, were similar or lower to those used in this study for the three hill breeds.

The R2 retrotype, containing only enJSRV-18 and the most common world wide [17], was found in the Herdwick and Rough Fell, but was absent from the Dalesbred. Only two retrotypes were abundant in the Dalesbred sample population, the R6 (enJSRV-18+enJS5SF16) and R7 (enJSRV-18+enJS5SF-16+enJSV-7). The R6 had been found typical of breeds north of the Mediterranean [17] and together with the R7 retrotype was found in several of the UK breeds Chessa *et al* analysed, including to a high level in the Scottish Blackface [17]. The SBAs of the northern horned sheep in the UK, including those of the Scottish Blackface, Swaledale and Dalesbred, as well as the Rough Fell, suggest there has been an earlier cultural association of the breeds [11], [12], [36], [37] and the close similarity in pattern of retrotypes between the Rough Fell in our study and that of the Scottish Blackface in [17] is interesting.

Very surprisingly, as shown in Table S1, our study revealed that the two rare retrovirus insertions (enJSRV-15 and enJSRV-16) are both present in the Herdwick and Rough Fell. The two were previously found to be common only in Texel breed populations [17], and were sufficiently rare to be excluded from the global retrotype classification scheme. One of the insertions (enJSRV-15) was also present in three Dalesbred individuals. The Texel Breed Society refers to the present day Texels originating in the late 19th century from crosses of the native breed (Pielsteert/pin-tail) on the Texel Island, north of the Netherlands, with Lincoln, Leicester and Wensleydale breeds [38]. Given that none of those UK breeds were found to have the rare insertion events in the study of Chessa *et al* [17], our data suggest there may have been some historical association between the original Pin-Tail ancestral population of Texel Island and the sheep populations in the north of England.

In total, the analysis of enJSRV insertions has enabled us to identify a clear genetic distinction between the three UK hill breeds of this study. In particular, their absence from a substantial proportion of Herdwicks highlights the breed retains features of a primitive genome, continuing to reflect old indigenous populations. The presence of the two rare enJSRVs also provides some genetic evidence to support local folklore concerning the origins of sheep in the region. Whilst the connection between the Herdwicks and Viking settlers is often highlighted [39], our data clearly extend this association also to the Rough Fell breed.

### Analysis of SNPs

Several SNP analyses have explored patterns of genetic variation amongst sheep breeds across the world [18], [19], [26], [40]. In this study, we have used SNPs to complement other approaches and have limited our analysis of variation at SNP loci to some of those for which a gene-phenotype association has already been defined.

### Susceptibility to Maedi-Visna lentivirus infection – TMEM154

The Maedi-Visna (MV) ovine lentivirus causes an incurable slow acting disease affecting millions of sheep worldwide with massive economic and welfare impacts [41]–[43]. In recent studies, polypeptide variants encoded by haplotypes of the ovine gene *TMEM154*, were found to be associated with MV infection [25], [26]. The presence of glutamate (E) at position 35 in the ancestral, full length version of the protein was associated with increased susceptibility to the lentivirus, whereas lysine (K) at position 35, or deletion mutants, were associated with reduced susceptibility.

Table 2 gives the frequencies of the *TMEM154* 35E and the *TMEM154* 35K alleles in Herdwick, Rough Fell and Dalesbred sheep. In each breed, the frequency was found to be higher for the allele corresponding to reduced susceptibility.

**Table 2:** Genotype classes and frequencies of alleles for SNP locus (MV lentiviral susceptibility).

	Herdwick	Rough Fell	Dalesbred
Allele A	7	11	11
Heterozygous	8	6	4
Allele G	4	2	4
Total	19	19	19
Frequency Allele A	0.58	0.74	0.68
Frequency Allele G	0.42	0.26	0.32

Allele A, homozygosity for *TMEM154* 35K; Allele G, homozygosity for *TMEM154* 35E.

Heaton and co-workers recently reported the average frequency of the highly susceptible *TMEM154* alleles be 0.51 across 74 breeds world-wide [26] with >25% of those analysed with a frequency above 0.8, indicating a significant proportion of sheep globally could be at risk from MV infection. Our data, with frequencies for the susceptible allele ranging from 0.26 to 0.42, suggest the hill breeds represent populations with a lower than average risk of infection.

Whilst there is evidence to suggest the host/viral interactions are complex, with host genetic resistance conditional [25] and many factors likely to affect the relative risk of infection, including viral genetic adaptations to host defences [44], the prevalence of less susceptible alleles of *TMEM154* in any breeding population offers an important new route to eventual eradication of the disease.

### Reproductive performance – PRLR

The prolactin receptor is a member of the large superfamily of class 1 cytokine receptors and is known to affect important agronomic traits in livestock including growth and reproductive performance, such as described in [27], [28]. Recently, a SNP in the region of the *PRLR* gene was identified as a region of positive selection in the sheep genome [19] and Table 3 shows the data for the same SNP analysed in the three hill breeds. A clear difference is revealed between the Herdwick, compared to the Dalesbred and Rough Fell. The ‘C’ allele of this locus is present at a frequency of 0.45 in Herdwick, whereas in Dalesbred and Rough Fell it is comparatively rare (with frequencies of 0.1 and 0.03 respectively). Further studies will be required to understand the implications of these data and which aspects of *PRLR* function are reflected in the breed differences.

**Table 3:** Genotype classes and frequencies of alleles for SNP locus OAR16_41943180 (reproductive traits).

	Herdwick	Rough Fell	Dalesbred
Allele C	4	0	1
Heterozygous	9	1	1
Allele A	6	17	13
Total	19	18	15
Frequency Allele C	0.45	0.03	0.10
Frequency Allele A	0.55	0.97	0.90

### Presence of horns – RXFP2

Sexual weaponry, within the context of horns [29], is likely to play an important role in competitiveness within breeding sheep populations and has been studied extensively in the primitive, feral Soay sheep of St Kilda, Scotland [29], [45], [46].

In the three hill breeds in this study, there is a marked phenotypic difference in occurrence of horns between the breeds. The males and females of the Dalesbred and the Rough Fell sheep have prominent horns and there is an absence of either polled animals, or those with deformed, vestigial horned structures, defined as ‘scurs’ in the Soay literature [29]. Herdwicks have different phenotypes, in that males can be horned, polled, or have scurs, but females must be hornless and can fail approval if there is any sign of horn structures however minimal. According to local literature on the Herdwick breed, this strict sexual dimorphism for the development of horns and the incidence of polled females has greatly increased in more recent years [39].

A genome wide association study enabled Johnston *et al* [29] to identify the *Horns* locus as likely to be the gene encoding the Relaxin/insulin-like family peptide receptor 2. Recent analyses of a large number of sheep breeds from Kijas *et al* [19] confirmed the association of SNPs close to *RXFP2* were linked to the presence and type of horns.

Table 4 illustrates the clear difference at the SNP locus between the three breeds in this study. All of the Herdwick individuals were homozygous for one allele, whereas all the Rough Fell and most of the Dalesbred individuals were homozygous for the opposing allele. As yet, the functional implications of the allelic differences in the breeds are unknown, particularly given that only rams have been sampled and far greater numbers of both females and males need to be analysed. A future study involving detailed characterisation of *RXFP2* alleles by sequencing in the three breeds will be informative, and hopefully will also provide new insights into the genetics underpinning the full range of horn phenotypes found in Herdwick rams.

**Table 4:** Genotype classes and frequencies of alleles for SNP locus OAR10_29511510 (horn associated).

	Herdwick	Rough Fell	Dalesbred
Allele T	0	19	11
Heterozygous	0	0	7
Allele C	19	0	0
Total	19	19	18
Frequency Allele T	0.00	1.00	0.81
Frequency Allele C	1.00	0.00	0.19

### Analysis of microsatellites

Molecular characterization of European sheep breeds using microsatellites has most recently focused on large-scale comparisons, such as the study of 57 breeds across Europe and the Middle East by the Peter and coworkers of ECONOGENE Consortium [16], 29 breeds across Europe by Lawson Handley *et al* [15] and 32 breeds of northern Europe by Tapio *et al* [22]. Microsatellites have also been used to great effect in exploring allelic associations with environmental variables to provide indications of adaptations to local conditions in which breeds are farmed [47]. In this study we used a subset of the microsatellites developed for the large-scale comparisons to gain a first insight of the three hill breeds.

**Figure 3:**
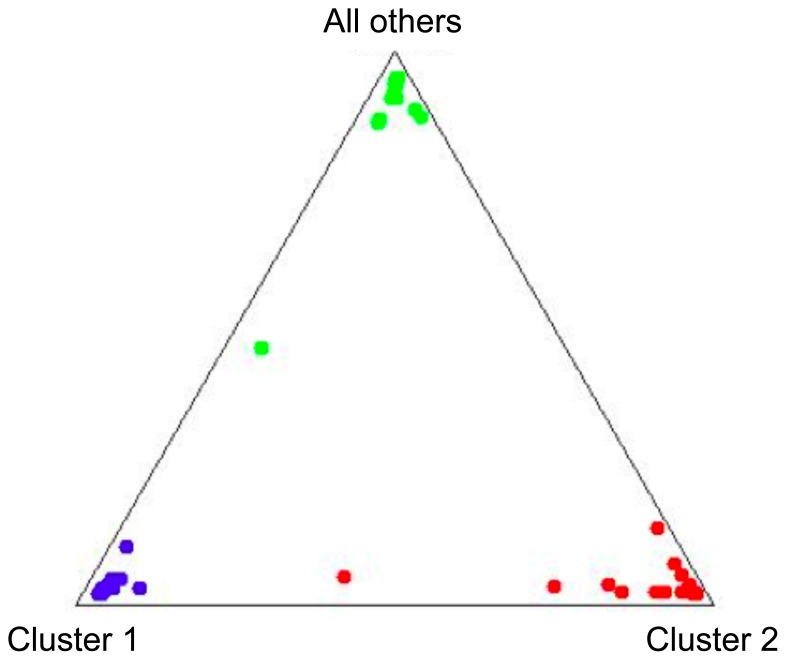
Analysis of microsatellite data. The microsatellite data were analysed with Structure; the most likely number of populations to explain the data was for K = 3 (−1525.1). Rough Fell corresponds with cluster 1, Dalesbred with cluster 2 and Herdwick with cluster 3 (all others).

The data were analysed with Structure and as shown in Figure 3 and Table 5, the triangle plot very closely agrees with the known breed of the individuals and the clusters of individuals. Rough Fell corresponds with cluster 1, Dalesbred with cluster 2 and Herdwick with cluster 3. Only two individuals separated from members of the same breed, a Dalesbred and a Herdwick, which Structure indicated may have received alleles from a Rough Fell individual at a grandparental generation. Pairwise *F*_ST_ between Herdwick and Rough Fell was found to be 0.1435, between Herdwick and Dalesbred to be 0.1562, and between Dalesbred and Rough Fell was 0.1817.

**Table 5:** The proportion of membership of each of the three breeds Rough Fell, Dalesbred and Herdwick in each of the three clusters inferred by Structure shown in Figure 3.

	Cluster 1	Cluster 2	All others
Dalesbred	0.069	0.906	0.025
Herdwick	0.046	0.02	0.934
Rough Fell	0.966	0.14	0.02

Several studies have used microsatellites for molecular characterization of geographically proximate breeds. For example, a pairwise *F*_ST_ of <0.1 was found for each of three breeds, Blond-faced Latxa, Black-faced Laxta and Rubia del Molar, farmed in directly abutting regions in Northern Spain [20]. In the central Volga area, pairwise *F*_ST_ values for three of the four breeds closest to one another (Estonian Blackhead, Whitehead and Finnsheep) ranged from 0.018 to 0.061, with the fourth (Ruhnu), located in the same region, displaying a greater pairwise *F*_ST_ at 0.173 [24].

It is possible that the level of genetic differentiation between breeds that we have observed reflects our sampling of unrelated breeding rams. Each of these were formally registered by the SBAs to conform to their phenotypic breed standards. However, we have only used a subset of microsatellite markers in this first analysis and a lower sample size than the other studies and that together with the different sampling methods used makes direct comparisons difficult.

No significant inbreeding, calculated using FStat, was revealed in any of the three breeds we studied, shown as the heterozygote deficiency within populations, per marker per breed in Table S3. The average local inbreeding coefficient (*F*_IS_) is weakly positive in all cases, with the lowest value of 0.059 in Herdwick and highest in Rough Fell (0.162). This suggests that the current informal breeding practices supported by the SBAs is helping to avoid the production of highly related breeding rams and maintains diversity within the genetic pool of each breed.

## Conclusions

Evidence for the genetic distinctiveness of the Herdwick, Dalesbred and Rough Fell breeds has been provided using a combination of DNA-based tools applied to registered unrelated rams conforming to breed standards. Our findings will help to inform national conservation strategies to ensure the genetic resources of these locally adapted hill breeds are protected. In particular, the unique feature of the primitive genome in the Herdwick population suggests the continued existence of rare gene pools that may provide useful adaptive fitness traits to increase the sustainability of livest9xls0ock agriculture in a changing climate.

## Supporting Information

File S1 **PCR details.**
(DOCX)

Table S1 **Data set of enJSRV insertions per individual of each breed.**
(XLSX)

Table S2 **Genotypes at 3 SNP loci.**
(XLSX)

Table S3 **Genotyping of sheep using microsatellites.**
(XLSX)

Table S4 **Inbreeding coefficients per marker per breed.**
(XLSX)
