# Supplementary material for: Genetic Distinctiveness of the Herdwick Sheep Breed and Two Other Locally Adapted Hill Breeds of the UK
Source: PLoS One. 2014 Jan 29;9(1):e87823. doi: 10.1371/journal.pone.0087823 (PMC3906253; doi:10.1371/journal.pone.0087823)
Supplement: File S1 — PCR details. (DOCX) [file pone.0087823.s001.docx]

**File S1 PCR details**

*Retrovirus insertion analysis*

The primers used to detect the common retrovirus insertions enJSRV-18, enJSRV-7, enJS5F16, enJSRV-8, and the rare insertion events enJSRV-15, enJSRV16 were as described by Chessa *et al* [17].

The loci were amplified using the following Touchdown PCR

| Cycles | Temperature (°C) | Time | Note |
| --- | --- | --- | --- |
| Denature | 95 | 10 min |  |
| Touchdown | 95 | 15 sec |  |
| 8 cycles | 61 | 30 sec | decrease 0.5°C/cycle |
|  | 72 | 30 sec |  |
| 30 cycles | 95 | 15 sec |  |
|  | 57 | 30 sec |  |
|  | 72 | 30 sec |  |

Following thermal cycling the products were diluted with water and added to HiDi Formamide containing Liz600 size markers. The mixtures were then electrokinetically injected into an ABI3730 instrument, and the data collected and analysed in GeneScan software.

*SNP analysis*

Three SNP loci were analysed using the KASPar reagent system (LGC Genomics Ltd., Units 1&2, Trident Industrial Estate, Pindar Road, Hoddesdon, Herts EN11 0WZ).

The primers used were:

| TMEM154E35KL | GTATGTGTTYCCACAGGAGAGGAG |
| --- | --- |
| TMEM154E35KFA | GAAGGTGACCAAGTTCATGCTGGGCACGTCTCCTGACAGTTT |
| TMEM154E35KVG | GAAGGTCGGAGTCAACGGATTGGGCACGTCTCCTGACAGTTC |
|  |  |
| OAR10_29511510FC | GAAGGTGACCAAGTTCATGCTTCATACACCTAGAAAACGTCCTGC |
| OAR10_29511510FT | GAAGGTCGGAGTCAACGGATTATCTCATACACCTAGAAAACGTCCTGT |
| OAR10_29511510RL | TCAGAATCTTTTCACGCAGAAGAC |
|  |  |
| OAR16_41943180FL | GTGGTGGTAGATATGTATTTCATTCCA |
| OAR16_41943180RA | GAAGGTGACCAAGTTCATGCTTCACATCCCTAAATGAGATGAATATGTAT |
| OAR16_41943180RC | GAAGGTCGGAGTCAACGGATTTCACATCCCTAAATGAGATGAATATGTAG |

The primers for TMEM154 were designed against sequences described by Heaton *et al* [25] whilst the other two were designed against sequences from Kijas *et al* [18].

The SNP locus OAR10_29511510 is within the eleventh intron of the gene *RXFP2* and was selected because of its association with the development of horns [29] was shown by Kijas *et al* [19] to be in a region that is under strong selection.

OAR16_41943180 is 207 kb at 3’ to the gene encoding sperm flagellar protein 2 (complementary strand) and 307 kb at 5’ to the gene encoding the prolactin receptor precursor (PRLR). The SNP was selected because it was shown by Kijas *et al* [19] to be in a region that is under strong selection, near to *PRLR* known to influence reproductive traits.

The loci were amplified using a touchdown procedure:

| Cycle | Temperature (°C) | Time | Note |
| --- | --- | --- | --- |
| Activation | 94 | 15 min |  |
| 10 cycles | 94 | 20 sec |  |
|  | 65-57 | 20 sec | decrease 0.8°C/cycle |
| 26 cycles | 94 | 20 sec |  |
|  | 57 | 60 sec |  |

The products were visualized in an Applied Biosystems 7900 and scored with the Applied Biosystems SDS software.

*Microsatellite analysis*

The following primers were used in the SSR analysis; these primers are in common between two previous studies, Peter *et al* [16] and Tapio *et al* [23].

| BM8125F | Fam-CTCTATCTGTGGAAAAGGTGGG |
| --- | --- |
| BM8125R | GGGGGTTAGACTTCAACATACG |
|  |  |
| MAF214F | Fam-GGGTGATCTTAGGGAGGTTTTGGAGG |
| MAF214R | AATGCAGGAGATCTGAGGCAGGGACG |
|  |  |
| MAF65F | Fam-AAAGGCCAGAGTATGCAATTAGGAG |
| MAF65R | CCACTCCTCCTGAGAATATAACATG |
|  |  |
| McM527F | Fam-GTCCATTGCCTCAAATCAATTC |
| McM527R | AAACCACTTGACTACTCCCCAA |
|  |  |
| OarAE129F | Fam-AATCCAGTGTGTGAAAGACTAATCCAG |
| OarAE129R | GTAGATCAAGATATAGAATATTTTTCAACACC |
|  |  |
| OarCP34F | Fam-GCTGAACAATGTGATATGTTCAGG |
| OarCP34R | GGGACAATACTGTCTTAGATGCTGC |
|  |  |
| OarFCB128F | Fam-CAGCTGAGCAACTAAGACATACATGCG |
| OarFCB128R | ATTAAAGCATCTTCTCTTTATTTCCTCGC |
|  |  |
| OarFCB304F | Fam-CCCTAGGAGCTTTCAATAAAGAATCGG |
| OarFCB304R | CGCTGCTGTCAACTGGGTCAGGG |
|  |  |
| OarHH47F | Fam-TTTATTGACAAACTCTCTTCCTAACTCCACC |
| OarHH47R | GTAACTTATTTAAAAAAATATCATACCTCTTAAGG |
|  |  |
| OarVH72F | Fam-CTCTAGAGGATCTGGAATGCAAAGCTC |
| OarVH72R | GGCCTCTCAAGGGGCAAGAGCAGG |

The reactions were set up in Sarstedt 384-well plates and cycled in ABI9700 thermal cyclers (Applied Biosystems). The PCR cycling employed a touchdown procedure and the conditions were:

| Cycle | Temperature (°C) | Time | Note |
| --- | --- | --- | --- |
| Denature | 94 | 10 min |  |
| Touchdown | 94 | 15 sec |  |
| 8 cycles | 60 | 30 sec | decrease 1.5°C/cycle |
|  | 72 | 30 sec |  |
| 30 cycles | 94 | 15 sec |  |
|  | 48 | 30 sec |  |
|  | 72 | 30 sec |  |

Following thermal cycling the products were diluted 1:200 with water, and then 1uL was added to 9uL HiDi Formamide containing Liz600 size markers (3uL markers + 1mL HiDi formamide). The mixtures were then electrokinetically injected into an ABI3730 instrument, and the data collected and analysed in GeneScan software.
